# Supplementary material for: Identification of photoperiod-induced specific miRNAs in the adrenal glands of Sunite sheep (Ovis aries)
Source: Front Vet Sci. 2022 Jul 22;9:888207. doi: 10.3389/fvets.2022.888207 (PMC9354845; doi:10.3389/fvets.2022.888207)
Supplement: Supplementary file 1 [file Data_Sheet_1.ZIP › Supplementary file/miRDeep2 file.zip!/miRDeep2 file/Novel_Structure/SP42A3/Novel_559.pdf]

Provisional ID : NC_019464.2_13541
Score total : 371.3
Score for star read(s) : 3.9
Score for read counts : 363.6
Score for mfe : 2.2
Score for randfold : 1.6
Score for cons. seed :
Total read count : 725
Mature read count : 694
Loop read count : 0
Star read count : 31

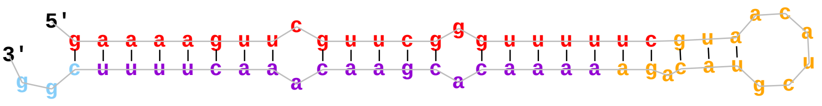

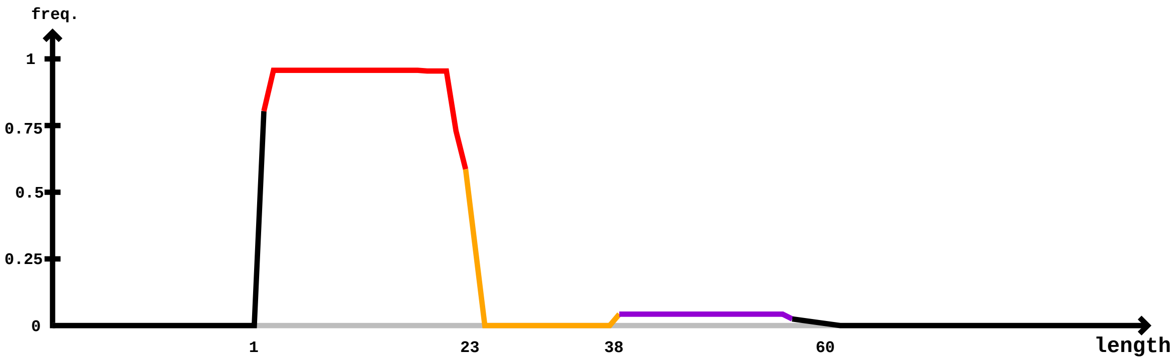

Mature **Star**

```
5'- auuaucauauuggguuggcugaaaaguucguucggguuuuucguaacaucguacagaaaaacacgaacaaacuuuucggccaacccaguauuucauucaucugcguuuuccu   -3'    obs
    auuaucauauuggguuggcugaaaaguucguucggguuuuucguaacaucguacagaaaaacacgaacaaacuuuucggccaacccaguauuucauucaucugcguuuuccu          exp
    ......((((((((((((((((((((.(((((.((((((((((......))).))))))).))))).))))))))))))))))))))).........................    reads    mm    sample
    ....................gaaaaguucguucgggu...........................................................................    2        0     seq
    ....................gaaaaguucguucggguuuu........................................................................    156      0     seq
    ....................gaaaaguucguucggguuuuuc......................................................................    425      0     seq
    .....................aaaaguucguucggguuuu........................................................................    7        0     seq
    .....................aaaaguucguucggguuuuu.......................................................................    104      0     seq
    ........................................................aaaacacgaacaaacuuu......................................    13       0     seq
    ........................................................aaaacacgaacaaacuuuu.....................................    18       0     seq
```
